# Presence and activity of Fibrinogen like protein 2 in platelets

**DOI:** 10.1371/journal.pone.0285735

**Published:** 2023-05-18

**Authors:** Izhack Cherny, Pinhas Hasin, Lital Kalich Philosoph, Yael Shahal-Zimra, Ronit Gurion, Esther Rabizadeh

**Affiliations:** 1 Hemato-Oncology Laboratory, Felsenstein Medical Research Center, Petah Tikva, Israel, Sackler Faculty of Medicine, Tel Aviv University, Tel Aviv, Israel; 2 Hematology Laboratory, Beilinson Hospital, Rabin Medical Center, Petah Tikva, Israel; 3 Institute of Hematology, Davidoff Cancer Center, Rabin Medical Center, Petah Tikva, Israel, Sackler Faculty of Medicine, Tel-Aviv University, Tel-Aviv, Israel; The University of Lahore, PAKISTAN

## Abstract

**Background:**

Fibrinogen-like protein 2 (FGL2) is a serine protease capable of converting prothrombin into thrombin (*i*.*e*., prothrombinase-like activity) while bypassing the classic coagulation cascade. It has been reported to be expressed by mononuclear blood cells and endothelial cells. There are multiple reports that FGL2 supports tumor development and metastasis. However, in the blood, the origin and functional significance of FGL2 has not been established.

**Objective:**

To determine if FGL2, a malignancy related enzyme, is present in platelets.

**Methods:**

Peripheral blood samples were collected in K_2_ EDTA tubes. Blood cells and platelets were separated and thoroughly washed to produce plasma-free samples. Procoagulant activity was measured in the cell lysates using a thrombin generation test or an adjusted prothrombin time (PT) test in plasma deficient of factor X. The findings were further supported by confocal microscopy, immunoprecipitation, flow cytometry, enzyme-linked immunosorbent assays and specific inhibition assays.

**Results:**

FGL2 protein was readily detected in platelets. Also, despite being expressed by lymphocytes, FGL2 prothrombinase-like activity was solely detected in platelet samples, but not in white blood cell samples. Quiescent platelets were shown to contain the FGL2 protein in an active form. Upon activation, platelets secreted the active FGL2 into the milieu.

**Conclusions:**

Active FGL2 is found in platelets. This suggests another role for the involvement of platelets in malignancies.

## Background

Fibrinogen-like protein 2 (FGL2) prothrombinase (50–60 KDa) [1] is capable of directly cleaving prothrombin into thrombin. It is a type II transmembrane glycoprotein (referred to as mFGL2), which is involved in the pathophysiology of multiple diseases, from microthrombosis to viral infections and cancer [2,3]. A second form of FGL2 (referred to as sFGL2, mainly produced by lymphocytes) is a potent immunosuppressor of the innate immune system, which is constitutively secreted by lymphocytes [4,5]. While the procoagulant activity of FGL2 has been reported in the blood in response to pathologic stimuli, its specific origin in the blood is not clear.

Understanding the cellular origin of FGL2 procoagulant activity in the blood is important since FGL2 is engaged in a wide range of biological processes involving microthrombosis [6–17]. Moreover, accumulating evidence established the significance of FGL2 in the biology of cancer by supporting tumor development and metastasis [18–28]. FGL2 was found to be highly upregulated in various solid tumors, including liver, renal, colon, breast, lung, gastric, esophageal, and cervical cancers [21]. Correspondingly, the targeting of FGL2 has been consistently demonstrated to hold valuable therapeutic promise [18–20,22–24,26,29,30]. For instance, we have shown that knockdown of FGL2 in a prostate carcinoma cell line reduced subcutaneous xenograft tumor growth and angiogenesis in a SCID mice model *via* the involvement of FGL2 in the MAPK signaling pathway [20].

The basis for this study rested on our previous findings which had demonstrated that peripheral blood mononuclear cells-enriched (PBMC) samples from patients with non-Hodgkin’s lymphoma and cutaneous T-cell lymphoma hold elevated prothrombinase-like activity [31,32]. While FGL2 was established to be expressed and secreted by the lymphocytes, (in addition to endothelial cells and macrophages) [2], PBMC samples do not contain endothelial cells or macrophages, but unavoidably contain all types of white blood cells in different amounts, including platelets. Therefore, we sought to validate the origin of the procoagulant activity in blood cells.

In this study we identify the origin and the low, yet significant, coagulant activity in PBMC samples [31,32]. Surprisingly, detected activity was solely correlated with the number of platelets in the samples. Platelet-free samples lacked coagulant activity. We provide evidence for the presence and activity of FGL2 in the platelets. Because platelets are activated at the interface of thrombosis, immune response and cancer [33,34], our study may also provide a fresh look into platelet-cancer relationships.

## Methods

### Study population

Peripheral blood samples were collected from healthy volunteers (n = 65) in the Rabin Medical Center, Beilinson. The study was approved by the hospital’s Institutional Review Board. A written informed consent was obtained from all volunteers.

### Peripheral blood cells isolation

Whole blood samples were collected in K_2_ EDTA tubes. White blood cells and platelets were isolated using UNI-SEP tubes (Novamed, Israel) according to manufacturer instructions. In order to prevent platelet activation, CTAD solution was added in all wash steps.

### Platelet isolation

Platelet-rich plasma (PRP) was obtained by centrifugation (200 × g, 20 min, 20°C). The upper fraction of the PRP (*ca*. half volume) was aspirated and thoroughly washed with calcium and magnesium-free PBS (4500 × g, 5 min, 20°C, twice). The composition of samples was determined using automated differential hematology analyzers routinely used in the clinical hematology laboratories (XN-1000, Sysmex or Advia 2120i, Siemens). Predetermined amounts of platelets were aliquoted, centrifuged (7500×g, 3 min, 20°C), and stored as dry pellet at -80⁰C until analysis. The storage of the samples was verified to not affect the activity.

### Thrombin generation assay

Activity assays were performed as previously described [31]. Briefly, predetermined amounts of cells were aliquoted, homogenized, mixed with 10 mM human prothrombin (Stago, France) and incubated for 30 min at 37°C to allow thrombin generation. The reaction was terminated, and the amount of generated thrombin was measured using a chromogenic substrate simulating the fibrinogen proteolysis site (S-2238TM; Chromogenix, Italy) and calculated according to a standard curve comprised of human thrombin with known international unit (IU) concentrations (Omrix, Israel).

### Prothrombin time assay

Prothrombinase activity in peripheral blood cell samples was measured by an automated coagulation analyzer (ACL-TOP 500; IL, Italy) based on an extended prothrombin time (PT) test. Cell samples were suspended, homogenized and analyzed in commercial factor X deficient plasma (FXDP) (Stago, France or IL, Italy). The homogenization process was verified to not affect activity. According to the manufacturer, FX levels are less than 1% and 3% in the ‘Stago’ and ‘IL’ deficient plasma, respectively. The reaction was initiated by the addition of 100 μl thromboplastin reagent (RecombiPlasTin 2G (IL, Italy)) to 50 μl of the homogenized sample at 37°C.

Thrombin generation (TG) and PT methods measure different end products (*i*.*e*., fibrinogen-based substrate proteolysis rate *versus* fibrin clot formation time). We verified that the methods were comparable in healthy controls (n = 40). The samples included white blood cells and platelets, and aliquoted to include identical counts of mononuclear cells (1.5 × 10^6^). Comparison between the measurements showed that the clotting time inversely correlated with thrombin generation activity with high significance (τ = -0.44, p-value <0.0001) and strong linearity (*r* = -0.71) (Table 1 and S1 Fig). This confirmed that the two methods essentially reflect the same activity.

**Table 1:** Healthy study population and correlation to FGL2 activity.

**Number**	**65**
Gender: % males	67.7%
Age: median (range)	55 (23–74)
**Kendall Correlation coefficient (F074) between prothrombin time and subject/sample parameters:**	
Gender (male)	0.035
Age	0.075
Thrombin generation activity (n = 40)	-0.44***
Platelet count	-0.493***
White blood cells (total)^a^	-0.106
Neutrophils	0.001
Lymphocytes	-0.093
Monocytes	-0.038
Eosinophils	0.049
Basophils	-0.035

^a^ PBMC in the cohort having a fixed number of 1.5×10^6^ cells per sample.

*** *p*-value < 0.0001.

### Cell cultures

A PC3 cell-line, established from human prostate carcinoma, was cultured in F12 with 10% fetal calf serum. A CMK cell line, established from human acute megakaryocytic leukemia, was cultured in RPMI 1640 with 20% fetal calf serum.

### FGL2 cloning and overexpression in PC3 cells

Materials and method are described in supplementary methods section.

### Platelets activation

Activation of the platelets was performed using 4 μg/ml human type III collagen (Sigma-Aldrich, Israel), 4 μM ADP, or 250 μg/ml arachidonic acid (Helena Laboratories, USA). Activation was performed in PBS or FXDP (Stago, France).

### Immunoprecipitation

Platelet samples were thoroughly washed with PBS and sonicated in ice using a Vibra-Cell sonicator (Sonics and Materials, USA) (Tapered microtip, 40% amplitude, 2 sec ON time, 3 sec OFF, 12 cycles). Identical aliquots containing 2 × 10^7^ platelets were incubated with 1 μg of one of the following antibodies for 1 h at 4°C: monoclonal IgG_2a_ mouse anti-FGL2 (M01, clone 6D9, Abnova, Taiwan); or monoclonal rabbit anti-factor-X IgG_1_ (sc-81739 Santa Cruz Biotechnology, USA); or normal mouse IgG_2a_ (sc-3878, Santa Cruz Biotechnology, USA); or mouse IgG_1_ (sc-3877, Santa Cruz Biotechnology, USA). The lysed cells suspension was incubated with protein A/G-agarose beads (Santa Cruz Biotechnology, USA) for another hour at 4°C. The beads were washed four times with PBS supplemented with 0.1% tween-20, resuspended in sample buffer containing 2% SDS and 30 mM β-mercaptoethanol, and boiled for 3 minutes.

### Western blot

Materials and method are described in supplementary methods section.

### Enzyme-linked immune-absorption assay

Materials and method are described in supplementary methods section.

### Confocal microscopy

Glass cover slips were coated with 50 μg/ml fibrinogen (Sigma, Israel) for 2 hours at 37⁰C and kept overnight at 4⁰C. PRP were washed and suspended in pre-warmed PBS (37⁰C). Next, PRP was diluted 1:10 in PBS supplemented with 5 mM glucose and allowed to adhere to the fibrinogen coated wells for 10 minutes in 37⁰C. To activate platelets, 1 IU of Thrombin (Mallinckrodt Pharmaceuticals, USA) was added for another 40 minutes in 37°C. After washing with PBS, cells were fixed for 20 minutes with 3% paraformaldehyde in PBS and permeabilized by adding 0.5% Triton X-100 for another 2 minutes. Coverslips were thoroughly washed with PBS, blocked for 20 minutes with 2% bovine serum albumin (sigma, Israel) and immunostained. Coverslips were incubated with CellMask reagent,—plasma membrane stains (molecular probes life technologies) and different antibodies (according to manufacturer instructions) for 30 minutes at room temperature: mouse monoclonal anti-FGL2, mouse monoclonal anti Factor X, FITC-conjugated F(ab`)_2_ anti-mouse (Jackson Immunoresearch Laboratories, USA). The slides were washed with PBS, mounted and photographed using a Zeiss-LSM-510 confocal laser-scanning microscope (CLSM; Carl Zeiss MicroImaging; Plan-Neofluar 25× objective).

### Flow cytometry

The presence of FGL2 in platelets and white blood cells was analyzed by flow cytometry. Peripheral blood samples obtained by venipuncture were collected in K_2_-EDTA anticoagulant and processed within three hours of collection. Blood aliquots were incubated with anti-CD45 PC5 (clone J.33 Beckman Coulter, USA) and anti-CD41 PE (clone 5B12 Dako, USA) as recommended by the manufacturer. Labeling the anti-FGL2 IgG_2a_ antibody (M01, clone 6DT, Abnova, Taiwan) or, as a negative control, normal mouse IgG_2a_ antibody (sc-3878, Santa Cruz Biotechnology, USA) with alexafluor488 was conducted manually using the APEX antibody labeling kit (Invitrogen, USA) according to the manufacturer’s directions. For intracellular detection, cells were permeabilized with the FIX & PERM kit (Thermo scientific, USA) according to the manufacturer’s directions. Two micrograms of each alexa fluor 488-labeled monoclonal antibodies were used. After 20 minutes incubation at room temperature, cells were washed using PBS. Acquisition was performed using a Beckman Coulter Navios multiparameter flow cytometer. A minimum of 30,000 events were collected. Leukocyte subsets were identified by CD45/SSc plots, and platelets were identified by CD41/low FSc plots. Data was analyzed using Kaluza v.1.3 Software.

### Inhibition of FGL2 activity assay using anti-NPG-12 antibody

The anti-NPG-12 IgG antibody [35] was incubated with protein samples for 2 hours at 37°C to maximize inhibition. Prothrombinase activity was measured immediately and after incubation. Normal rabbit IgG (R&D systems, USA) was used as a control.

### Inhibition of Factor X activity using Rivaroxaban

Rivaroxaban was extracted from Xarelto® 20 mg tablets (Bayer). A single tablet was dissolved in 1 ml Dimethyl sulfoxide, vortexed and diluted in calcium-free PBS to generate 2.6 μg/ml of the Rivaroxaban working solution. The exact active concentration was determined using a Liquid Anti-Xa assay kit (IL, Italy) based on a calibration curve generated with commercial Rivaroxaban calibrators (IL, Italy) and FXDP (IL, Italy) on an ACL-TOP 500 automated coagulometer (IL, Italy) according to manufacturer directions. Rivaroxaban was added up to a final concentration of 800 ng/ml in 200 ng/ml increments. Normal plasma pool (IL, Italy) served as a control. Coagulant activity was immediately measured using an extended PT assay.

### Statistical analysis

Analyses were performed using SAS 9.4 or Graph Pad Prism v.6 statistical software. The strength of the significance of the correlation between the sample parameters and clotting time (Table 1) was assessed using the Kendall rank correlation coefficient (τ). The associations between platelet count and activities were assessed by linear or power-regression correlation coefficients (r). Statistical significance was determined using Mann-Whitney or Student’s t-test where appropriate. P values ≤ 0.05 were considered statistically significant.

## Results

### Active procoagulant is located within peripheral blood platelets

**Fig 1:**
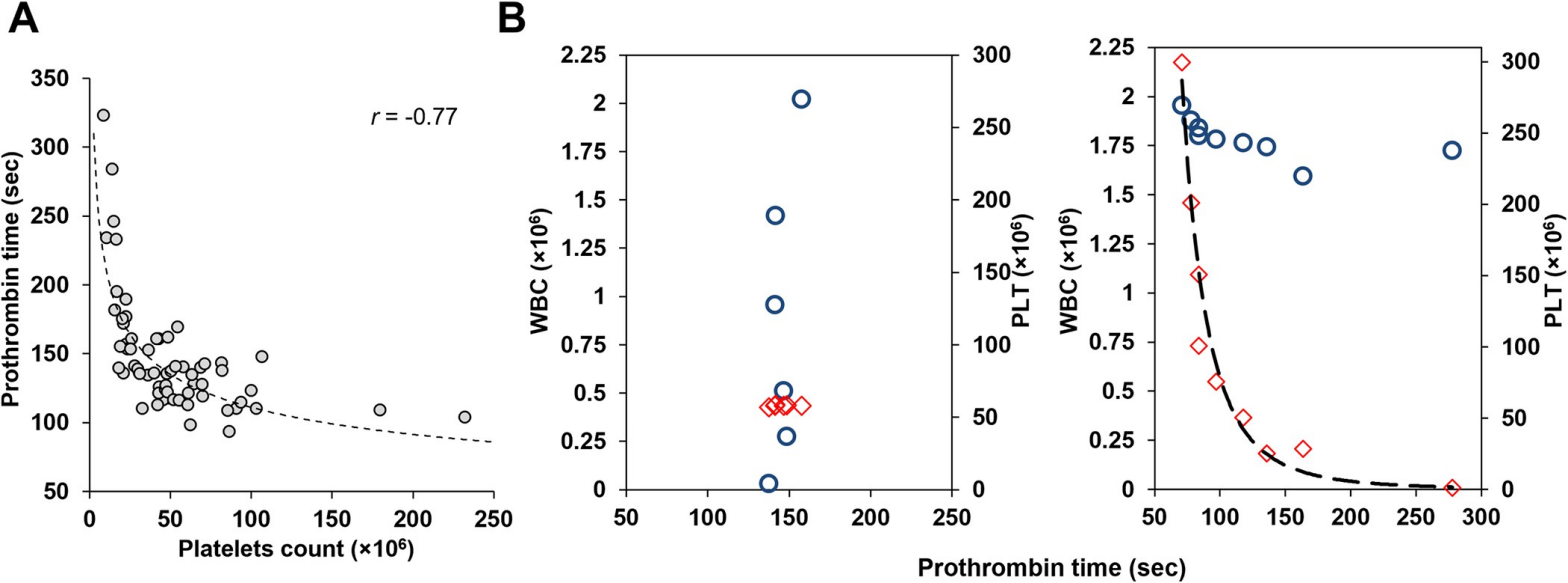
Prothrombinase activity in peripheral blood cell samples is dependent on the number of platelets in the sample. Analysis of Prothrombin Time (clotting time shortening) in samples of peripheral blood cell from healthy individuals homogenized in factor X deficient plasma. (**A**) Correlation (power-regression) between clotting time and the number of platelets in samples from healthy individuals. All samples include precisely 1.5×10^6^ mononuclear cells. (**B**) Correlation between Prothrombin time and counts of platelets (◇) or white blood cells (○). Platelets and white blood cells from a single donor were purified separately and then combined to generate different combinations. **Left panel B,** constant platelets count (58 × 10^6^) and increasing amounts of white blood cells (0.03–2×10^6^); **Right panel B**, similar white blood cells count (1.6–2×10^6^) and increasing amounts of platelets (0–1.5 × 10^6^).

Procoagulant activity was observed in PBMC samples [4,36]. To identify the specific cellular origin of the activity, correlation between the different cell type counts and activity (prothrombin time) in the samples were analyzed (Table 1). A significant and single correlation was detected between prothrombin time and the platelet count (τ = -0.49, p-value < 0.0001), together with a strong power-regression correlation (*r* = -0.77) (Fig 1A). To validate this finding, samples were prepared from a single donor and reconstituted to include similar concentrations of platelets with a gradient of white blood cells, and *vise versa* (Fig 1B). In agreement with the multiple sample observations (Fig 1A), the activities showed a complete dependency on the sample’s platelet count. Therefore, the origin of the observed coagulation activity appears to be in the platelets.

### The detected activity in platelets is prothrombinase

To verify that the PT assay in use is actually measuring a prothrombinase-like activity, rather than other enzymatic activities, several tests were performed.

**Fig 2:**
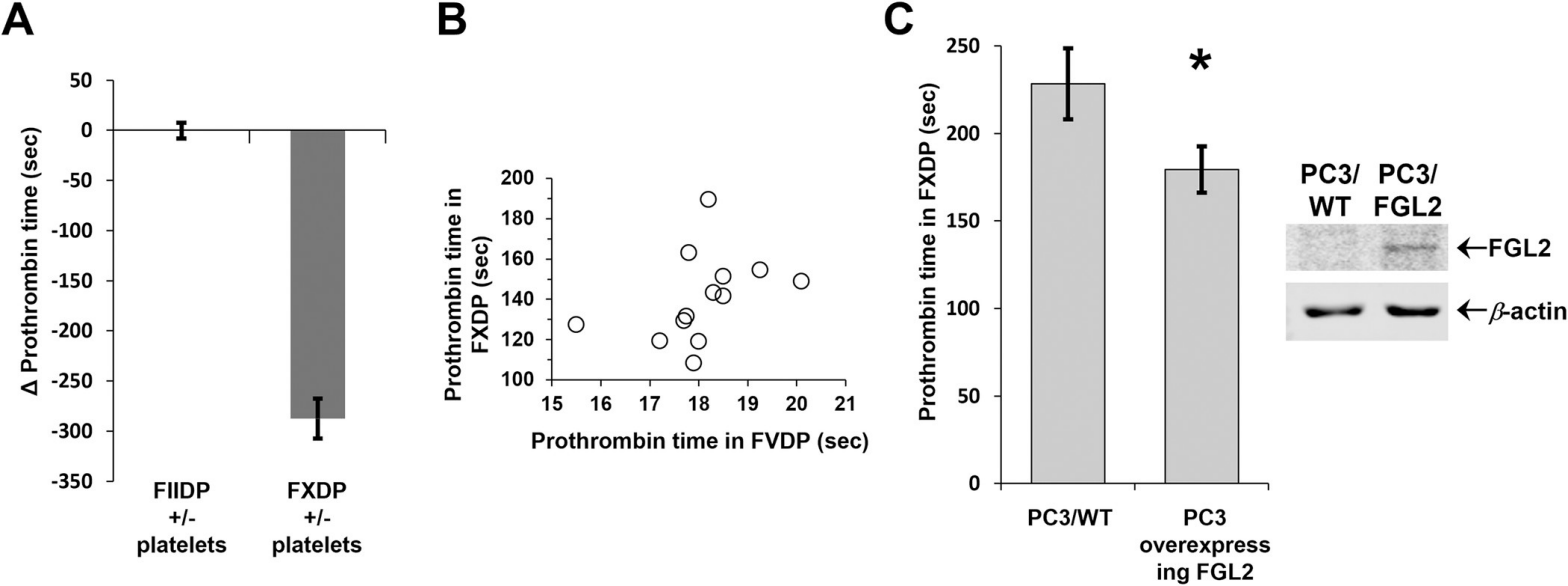
The detected procoagulant activity in platelets is prothrombinase. **(A)** A total of 2×10^8^ platelets were homogenized in factor-X deficient plasma (FXDP), or factor-II deficient plasma (FIIDP). Clotting was induced using the prothrombin time (PT) method. The time of clotting was recorded and compared to the time of clotting generated by samples of FXDP, or FIIDP only (without platelets). While both FIIDP samples elicit clotting after 97 seconds, regardless of the presence of platelets (Δ = 0±8 seconds), FXDP clotting time in the presence or absence of platelets was 109 and 397 seconds, respectively (Δ = 288±20 seconds). The measurements were performed in quadruplicates. Error bars represent standard deviation. (**B**) Twelve sample pairs of 10^8^ platelets were homogenized in FXDP versus FVDP and tested for prothrombin time activity. The correlation between prothrombin time generated by the samples in FVDP versus FXDP was weak (*r* = 0.38) and insignificant (*p* = 0.198). (**C**) Mean PT induced by PC3 wild type cells (228±19 sec) or FGL2 overexpressing cells (179±14 sec) homogenized in FXDP. Experiments were performed in triplicate. Error bars represent standard deviation (*p*-value = 0.01). FGL2 overexpression was confirmed by western blot analysis. β-actin was used as a control.

First, to rule out the possibility that prothrombin (factor II) or traces of thrombin (factor IIa) are driving the coagulation process (rather than prothrombinase), platelet samples were tested for their ability to induce clotting in factor II deficient plasma (FIIDP) (Fig 2A). Samples homogenized in FIIDP (Stago, France) failed to shorten the PT clotting time (0±8 seconds) in contrast to samples homogenized in factor X deficient plasma (Stago, France) where PT was shortened by 288±20 seconds.

Second, to rule out that variations in factor V (FV) levels regulate the levels of FGL2 activity, PT was tested in samples homogenized in FV-deficient plasma (FVDP) *versus* in FXDP (Fig 2B). FV acts as a cofactor of both FX and FGL2. Platelets are rich in FV and may therefore affect the PT test results. FV levels in the samples were sufficient to bring to near-normal PT values in FVDP (18±3 seconds). However, the correlation between clotting time generated by the samples in FVDP *versus* FXDP was weak (*r* = 0.38) and insignificant (p = 0.198). Therefore, prothrombinase activity rather than FV levels appears to determine the observed activity.

Last, to confirm that the PT assay is sensitive to FGL2 activity, yet independent of platelets or other unknown elements in the sample (such as traces of plasma, tissue factor, or debris of blood cells) the assay was performed using FGL2 expressed in an *in vitro* system. Recombinant FGL2 was overexpressed in a prostate carcinoma cell line (PC3). Prior to analysis, 1×10^6^ wild-type PC3 cells and FGL2 overexpressing PC3 cells were thoroughly washed with PBS, suspended in FXDP (Stago, France) and homogenized. The strain overexpressing FGL2 displayed significantly shorter clotting time compared to wild type cells (Fig 2C). This confirmed that FGL2 prothrombinase-like activity can be directly measured using the PT test.

### Identity of the platelet-borne prothrombinase

Two major prothrombinase enzymes are known: factor Xa and FGL2 [14,37]. To identify which prothrombinase is present in our samples, several methods were applied.

**Fig 3:**
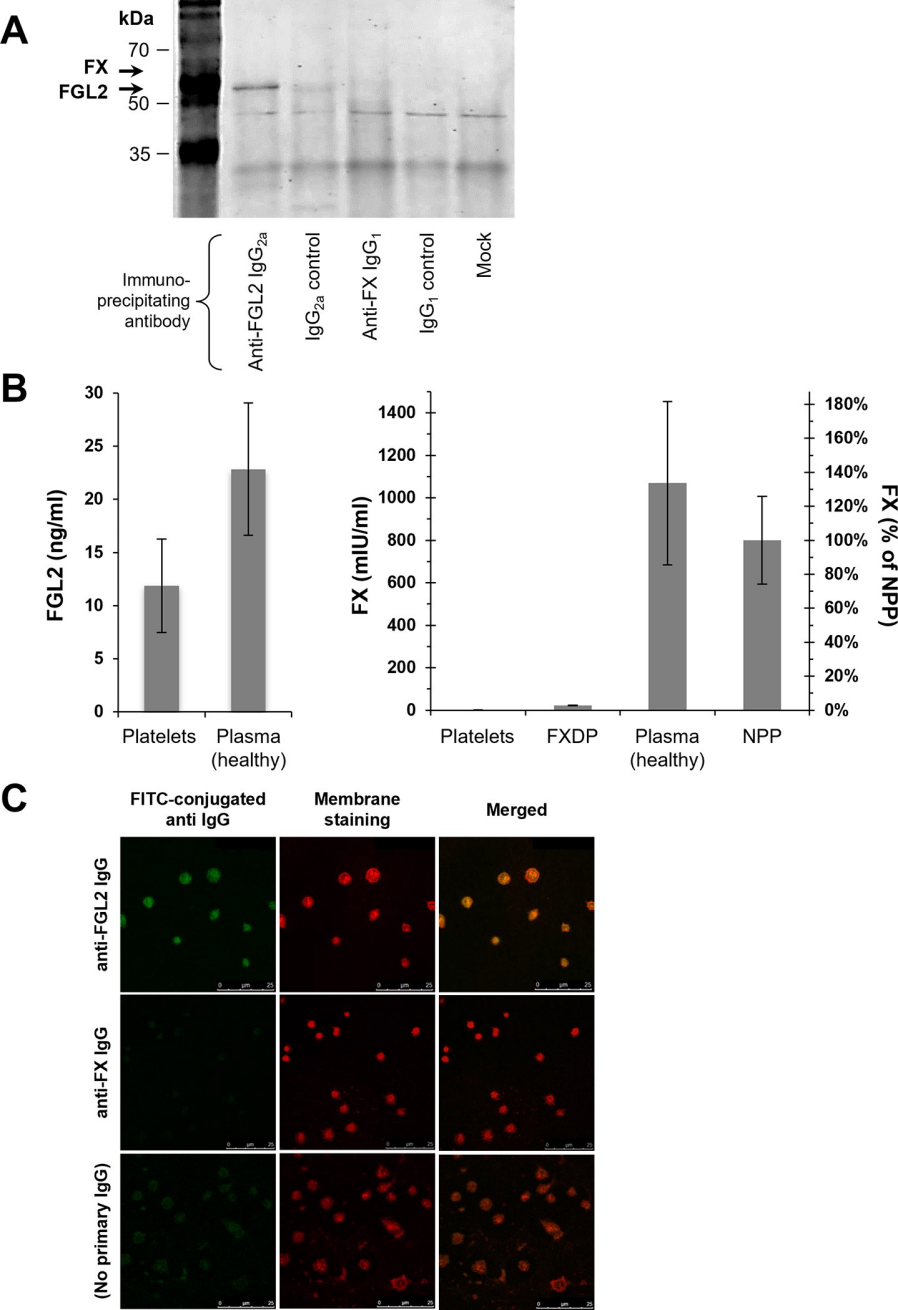
FGL2 is present in platelets. (**A**) Immunoprecipitation of FGL2 and FX from plasma-free peripheral blood samples. Immunoprecipitating antibodies and matched normal IgG subclasses are indicated. Mock represents nonspecific binding to protein A/G-agarose beads. Expected FX and FGL2 protein sizes are indicated by arrows. Only FGL2 was precipitated. (**B**) FGL2 and FX quantification using Enzyme-linked immunosorbent assays. Protein concentrations were determined in purified platelet samples (10^9^ platelets per sample, n = 11 healthy individuals), in plasma samples (n = 4 healthy individuals), in a normal pool plasma and in an FX-deficient plasma. Bars represent mean concentration. Error bars represent the standard deviation. (**C**) Confocal immunodetection of FGL2 or FX in freshly prepared non-stimulated platelets, assisted by FITC-conjugated secondary antibodies. Platelet membranes were fluorolabeled with CellMask deep red reagent. Background fluorescence (i.e., FITC-conjugated antibody only) is presented as well.

- *Immunoprecipitation*: FGL2 and factor X were immune-precipitated from identical aliquots derived from a single sample using monoclonal antibodies as well as matched normal IgG subclasses as controls. While FGL2 was readily immune-precipitated (in line with Marazzi *et*. *al*. [4]), factor X was not detected (Fig 3A).
- *Enzyme-linked immunosorbent assay*: Factor X levels were analyzed in samples containing 10^8^ purified platelets from healthy individuals (n = 7). Commercial factor X deficient plasma (Stago, France), commercial normal pooled plasma (IL, Italy) and healthy plasma samples served as a reference. Factor X level in the platelets was at least 1000-fold lower than healthy or normal pooled plasma levels and 25-fold lower than FX deficient plasma (Fig 3B). Given that the FX deficient plasma did not elicit clot formation, factor X level in platelet samples is negligible for the observed prothrombinase activity. FGL2 levels were measured in purified platelets (n = 11) and plasma (n = 4) from healthy individuals. FGL2 levels in the platelets and plasma were in the same order of magnitude (23±6 and 12±4 ng/ml, respectively) compatible with previous reported concentrations of FGL2 in the plasma [38–41].
- Confocal microscopy: Fresh platelet samples were fixed and permeabilized prior to immunostaining for FGL2 and FX. FGL2 was readily immune stained in the platelets (Fig 3C).

In summary, FGL2 was clearly detected in platelets. Our findings could not confirm a complete absence of FX in platelets.

### FGL2 is found in platelets

**Fig 4:**
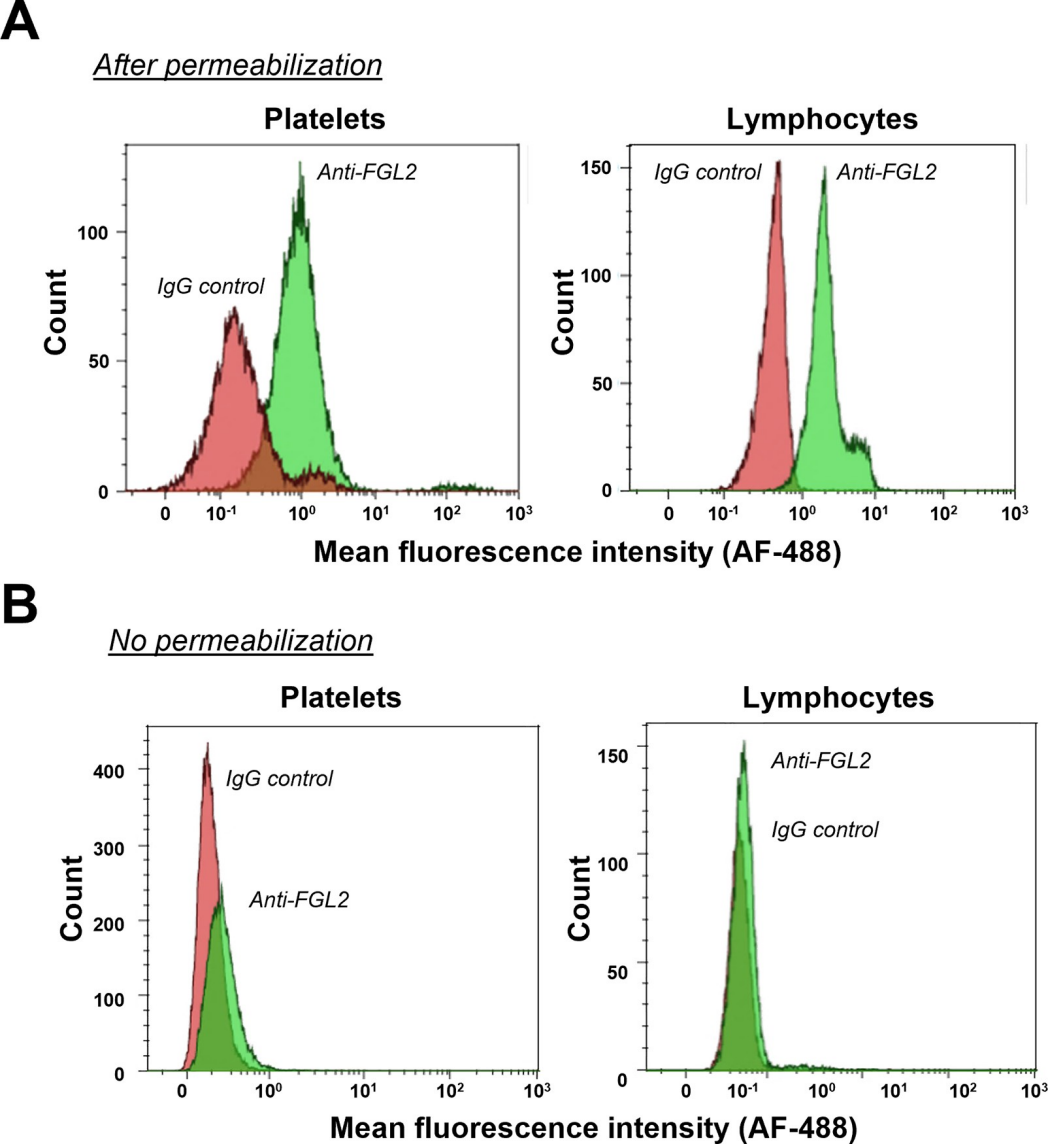
Flow cytometry detection of FGL2 in platelets and lymphocytes. The fluorescence intensity of IgG control-alexafluor 488 (Red) or FGL2-alexafluor 488 (Green) antibodies was detected in gated platelet using log SSC/FSC/CD41^+^ or in gated lymphocyte using CD45^+^/SSC plot following cell permeabilization (**A**) or intact cells (**B**).

To examine the localization of FGL2 in platelets, FGL2 was further examined by flow cytometry in whole blood samples, where the presence of FGL2 in lymphocytes served as a positive control. FGL2 protein was detected after permeabilization in lymphocytes and in platelets (Fig 4A). However, FGL2 could not be detected without permeabilization (Fig 4B). Taken together, this supports the finding that FGL2 is predominantly found intracellularly in quiescent platelets.

To gain insight into the origin of FGL2 in platelets, we tested a megakaryoblastic cell line, CMK. The CMK line is established from peripheral blood megakaryoblasts of a leukemia patient. Total protein and RNA were extracted from the CMK cell line. Western blot analyses showed no clear trace of FGL2 protein in megakaryoblasts (S2 Fig). Likewise, real-time PCR analysis was unable to detect traces of fgl2 mRNA in these cells.

### Platelets FGL2 is a procoagulant

**Fig 5:**
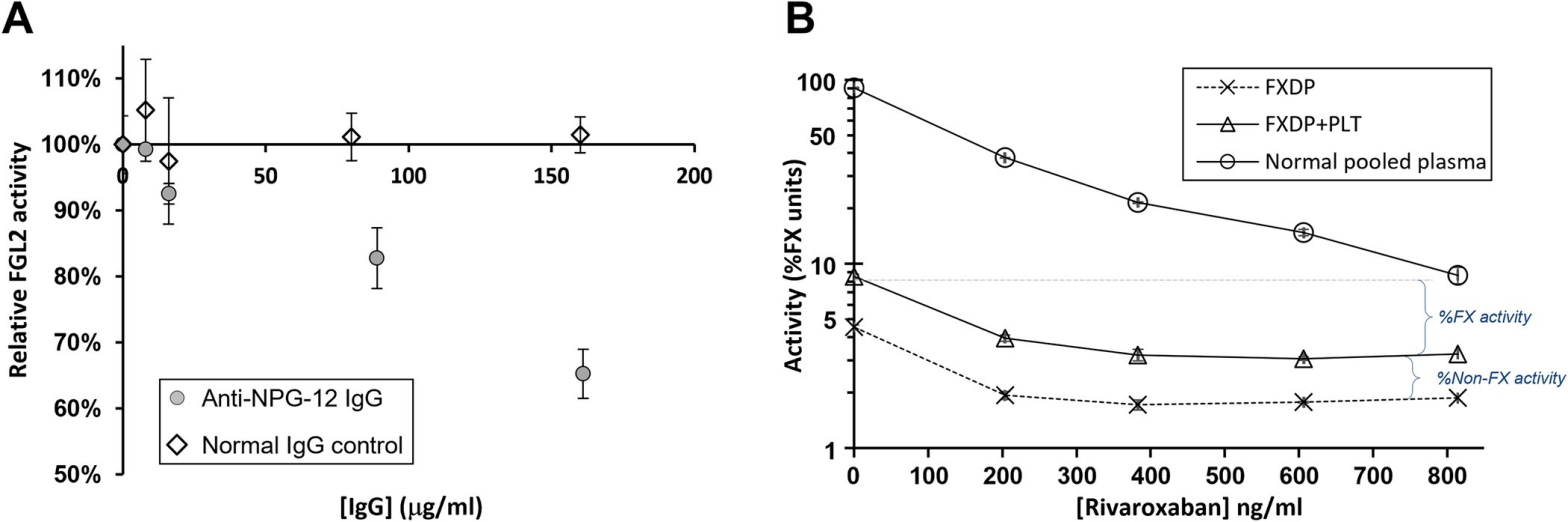
Validation of FGL2 activity in platelets. (A) inhibition of FGL2 activity by Anti-NPG-12 antibody. Platelets samples from peripheral blood were homogenized in FXDP (Stago, France) and incubated with increasing concentrations of Rabbit anti-NPG-12 antibody capable of specifically inhibiting FGL2 prothrombinase-like activity. Normal Rabbit IgG was used as a control. The resulting inhibition is expressed in percentages relative to the measured activity in the absence of antibody. Experiment was performed by three independent repeats (**B) Inhibition of FX activity by rivaroxaban.** Platelets samples from peripheral blood were homogenized in FXDP (IL, Italy) and incubated with increasing concentrations of rivaroxaban. FXDP and Normal pooled plasma were used as controls. The resulting inhibition is expressed in percentages of FX activity (log scale). The experiment was performed as two independent repeats. Error bars represent standard deviation.

To validate that platelet’s FGL2 exerted prothrombinase-like activity, direct inhibition assays of FGL2 and FX were performed (Fig 5).

Direct Inhibition of FGL2 was achieved using the Anti-NPG-12 antibody (Fig 5A). Anti-NPG-12 had previously been reported to inhibit FGL2 activity (at 100 μg/ml) in human umbilical vein endothelial cells (HUVECs) [35]. Accordingly, platelets homogenized in FXDP (Stago, France) were exposed to increasing concentrations of the antibody (up to 160 μg/ml). Prothrombin time was prolonged in correlation with the antibody concentration indicating up to a 35% decrease in activity (*p* < 0.05). Normal Rabbit IgG control showed no inhibitory effect. Accordingly, FGL2 contributed to the observed prothrombinase activity.

Direct inhibition of FX was achieved using rivaroxaban (Fig 5B). To effectively monitor the rivaroxaban’s inhibitory effect on FX, in an efficient way, platelets were homogenized in a commercial FXDP containing approximately 3% of residual FX (IL, Italy). Following exposure to increasing concentrations of rivaroxaban (up to 800 ng/ml), FX activity found in the FXDP reagent was effectively neutralized already at 200 ng/ml and completely at 380 ng/ml. However, in the presence of platelets, a consistent residual activity was observed that could not be inhibited even at 800 ng/ml rivaroxaban. The magnitude of the remaining non-FX activity (37% of initial activity) was in line with the fraction of activity that was inhibited by the Anti-NPG-12 antibody (Fig 5A), corresponding to *ca*.1.5%-2% FX activity units.

### Platelets secrete FGL2 upon activation

**Fig 6:**
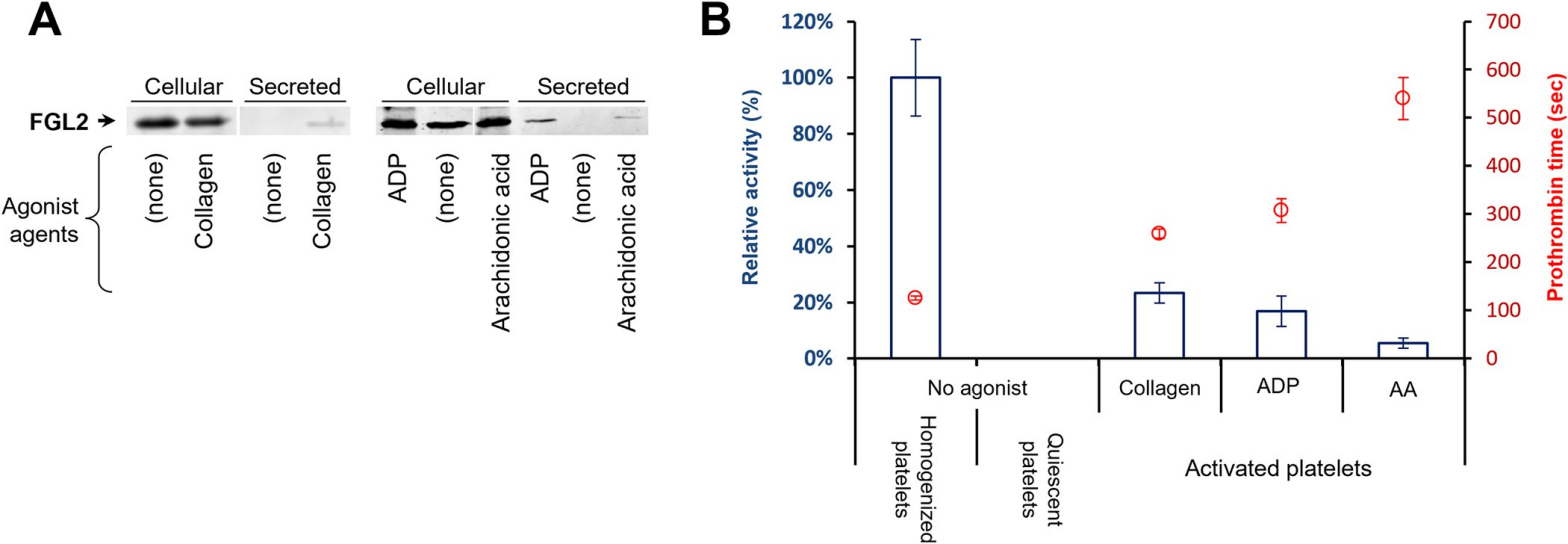
Platelets secrete active FGL2 upon activation. Identical and equal suspensions of 1×10^8^ platelets were treated with indicated agonists to stimulate platelets activation or maintain quiescence. The soluble fractions, containing the secreted proteins, were separated from the platelets fraction by centrifugation. **(A)** FGL2 was immunodetected by western blot in the supernatant of the activated platelets. **(B)** Activity was measured in the soluble fractions (containing the secreted proteins) by the prothrombin time assay. Clotting time shortening (represented by a red circle) was evident in the soluble fractions of the activated platelets, but not of the quiescent platelets. The column bars represent the corresponding activity, expressed as a percentage relative to the maximum activity obtained following complete homogenization of the sample (left bar). The experiment was performed in triplicates. Error bars represent standard deviation.

The extent of the biological significance for the presence of FGL2 in platelets depends on answering whether an active form of FGL2 is secreted after platelet activation. To this aim, the presence and prothrombinase-like activity of FGL2 were examined in the milieu of activated platelet samples (Fig 6). Isolated platelets were activated using the agonists collagen, ADP, or arachidonic acid (AA). Before activation, neither FGL2 antigen nor prothrombinase activity were detected in the supernatant liquid of quiescent platelets (prothrombin time was greater than 10 minutes). Following platelet activation, FGL2 antigen was readily detected in the liquid fraction of the activated platelets (Fig 6A) along with low but significant activity (Fig 6B). Activation using the strong agonist, collagen, resulted in a clotting time of 259 seconds, which corresponded to 23% of the maximum activity compared to complete homogenization of the sample (demonstrating a clotting time of 125 seconds). The weaker agonists (ADP and AA) were correspondingly less effective (demonstrating a clotting time of 307 and 540 seconds, respectively). Accordingly, the activity of FGL2 can be released into the blood upon platelet activation.

## Discussion

In this study, we provide evidence that (i) prothrombinase-like activity is present in the blood cells and is directly correlated with the number of platelets present in the sample (Figs 1 and 2); (ii) FGL2 is present in platelets (Figs 3 and 4); (iii) Platelet borne FGL2 is a prothrombinase (Fig 5); (iv) Platelet activation results in the secretion of active FGL2 into its milieu (Fig 6).

This is a major finding since it presents a new mechanism by which FGL2, an established procoagulant, immunomodulator and cancer associated protein, is introduced into the peripheral blood.

Further evidence supporting the occurrence of FGL2 in platelets may be obtained through searching in reported studies of high-throughput screenings of the platelet’s proteome and transcriptome. Both protein and RNA molecules of FGL2 were identified in an high-throughput platelet proteome screening study and in a paired-end next-generation RNA sequencing (RNA-seq) screening analysis of platelet transcriptome study [42,43]. However, the precise mechanism by which platelets acquire FGL2 is still unknown. FGL2 was not detected in a megakaryoblast cell line, thereby hindering our understanding of the physiologic significance of FGL2 in platelets. Since platelets uptake circulating proteins from the plasma [44,45], we speculate that FGL2 may also be taken up in the same manner. Yet, the exact mechanism is not known.

There are several implications for the identification of active FGL2 in platelets. First, previous studies pointing the procoagulant activity embedded in PBMC samples may have overlooked the presence or contribution of platelets. According to our study, PBMC lacks active form of prothrombinase.

Second, the discovery of FGL2 prothrombinase secreted by platelets is consistent with the role of platelets in the storage and secretion of coagulation factors and co-factors including factors V, XI, XIII, prothrombin (in their inactive forms), high molecular weight kininogens and polyphosphates [46]. With respect to the role of FGL2 in microthrombosis [8,9,16], our study suggests the involvement of platelets in mediating this process. We show that mere platelet activation had resulted in secretion of platelet-borne prothrombinase. It should be noted that the measured prothrombinase-like activity was rather low. Part of this activity may be contributed to other parameters that can be considered in a comprehensive kinetic analysis (*e*.*g*., although factor X could not be detected in platelets, it might be present at minuscule levels below the detection limit).

Third, the secretion of FGL2 by platelets suggests another source of tumorigenic factors. Platelets are multipurpose cell elements. Beyond their primary hemostatic role, platelets and their store of bioactive molecules provide the tumor microenvironment with vital support to sustain tumor proliferation and metastasis [34,47–51]. Indeed, overexpression of FGL2 in tumor cells and milieu have been related to increased proliferation and metastasis (either directly or mediated *via* generation of thrombin) [18–22,24,52]. Since accumulating evidence has demonstrated the potential of FGL2 as a therapeutic target and biomarker [18,20,24,29,31,32], the secretion of FGL2 by activated platelets at the cancer environment should also be taken into account in cancer research and therapeutics [33,53].

## Conclusions

FGL2, a prothrombinase and a malignancy-related enzyme, is present and secreted by activated platelets. As platelets are activated at the interface of thrombosis, immune response and cancer, our study may provide a fresh look into these relationships.

## Supporting information

S1 Fig Thrombin generation *versus* prothrombin time (PT) assays. Analysis of prothrombinase activity in lysed peripheral blood cell samples from healthy individuals. All samples include precisely 1.5×10^6^ mononuclear cells.
(TIF)

S2 Fig Analysis of FGL2 expression in peripheral white blood cells and Megakeryoblast cell line (CMK). Cells lysates were reduced and separated by SDS-PAGE. Presence of FGL2 and β-actin was analyzed by western blotting using polyclonal anti-FGL2 IgG.
(TIF)

S1 Raw images (PDF)

S1 Raw data (XLSX)

S1 File (DOCX)
