# Supplementary figures and images for: Presence and activity of Fibrinogen like protein 2 in platelets
Source: PLoS One. 2023 May 18;18(5):e0285735. doi: 10.1371/journal.pone.0285735 (PMC10194929; doi:10.1371/journal.pone.0285735)

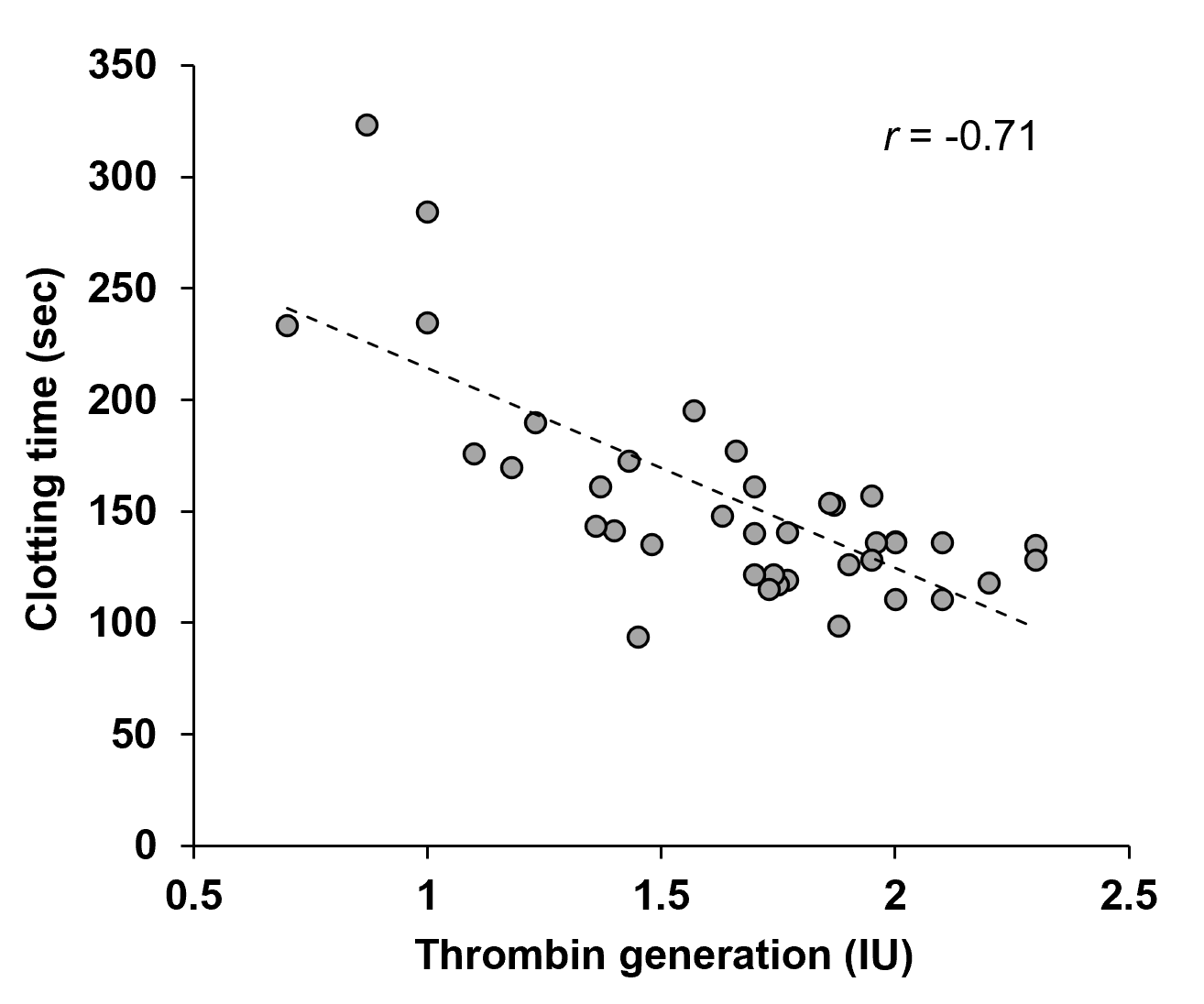

Supplement: S1 Fig — Analysis of prothrombinase activity in lysed peripheral blood cell samples from healthy individuals. All samples include precisely 1.5×10^6^ mononuclear cells. (TIF) [file pone.0285735.s001.tif]

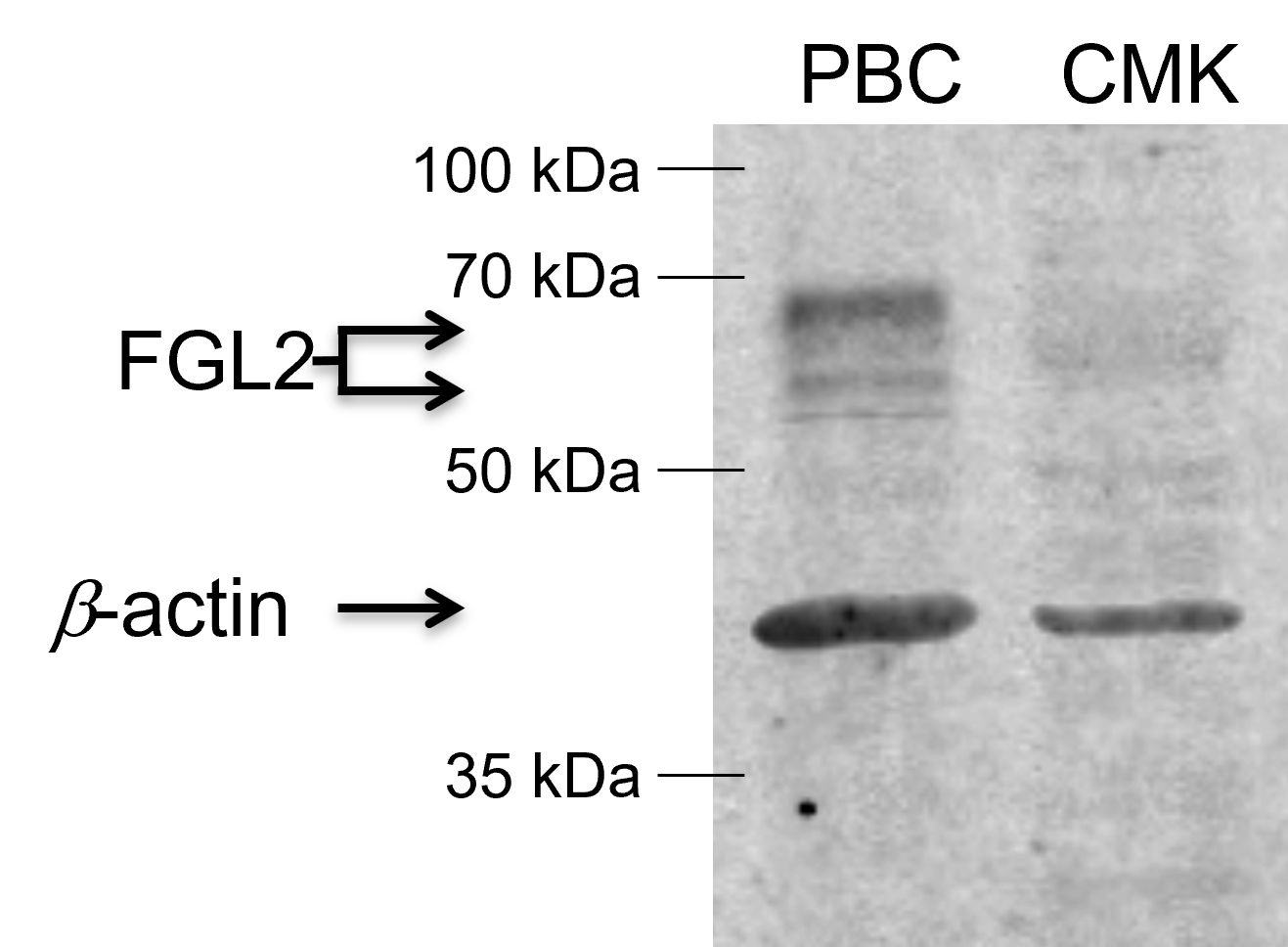

Supplement: S2 Fig — Cells lysates were reduced and separated by SDS-PAGE. Presence of FGL2 and β-actin was analyzed by western blotting using polyclonal anti-FGL2 IgG. (TIF) [file pone.0285735.s002.tif]
