# Supplementary material for: Presence and activity of Fibrinogen like protein 2 in platelets
Source: PLoS One. 2023 May 18;18(5):e0285735. doi: 10.1371/journal.pone.0285735 (PMC10194929; doi:10.1371/journal.pone.0285735)
Supplement: S1 Raw images — (PDF) [file pone.0285735.s003.pdf]

Figure 2 gel

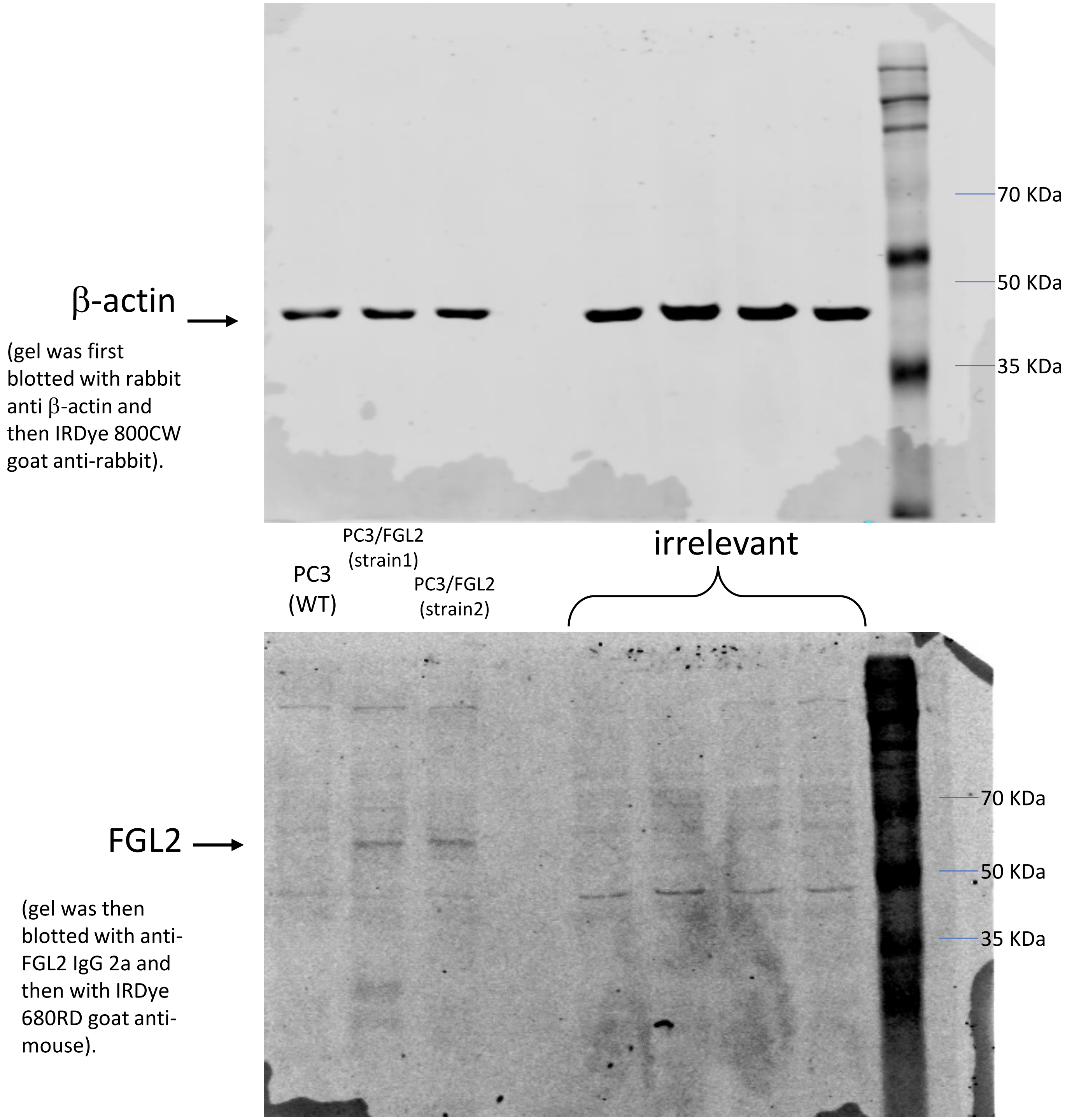

β-actin
(gel was first blotted with rabbit anti β-actin and then IRDye 800CW goat anti-rabbit).
70 KDa
50 KDa
35 KDa
PC3 (WT)
PC3/FGL2 (strain1)
PC3/FGL2 (strain2)
irrelevant
FGL2
(gel was then blotted with anti-FGL2 IgG 2a and then with IRDye 680RD goat anti-mouse).
70 KDa
50 KDa
35 KDa

Figure 3 gel

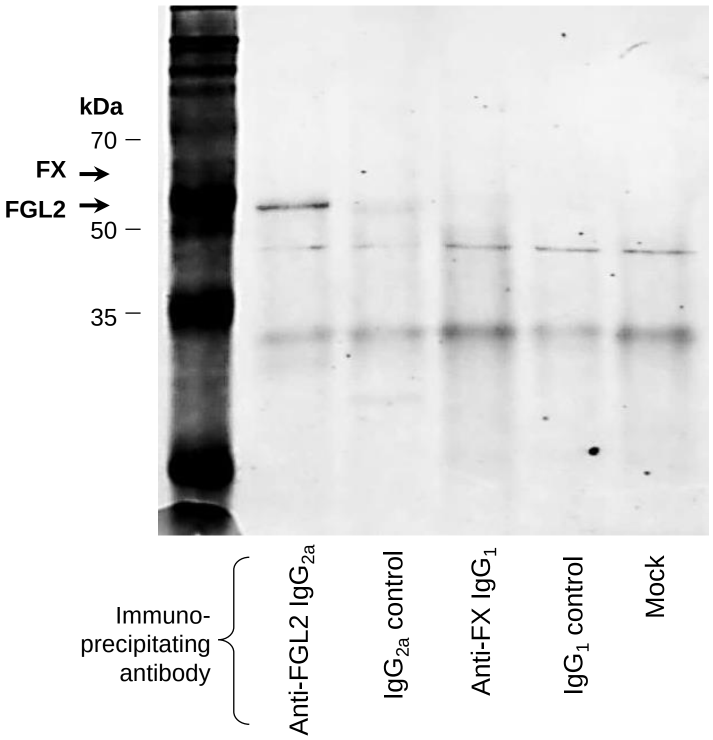
kDa
70
FX
FGL2
50
35
Immuno-precipitating antibody
Anti-FGL2 IgG2a
IgG2a control
Anti-FX IgG1
IgG1 control
Mock

Figure 6 gel

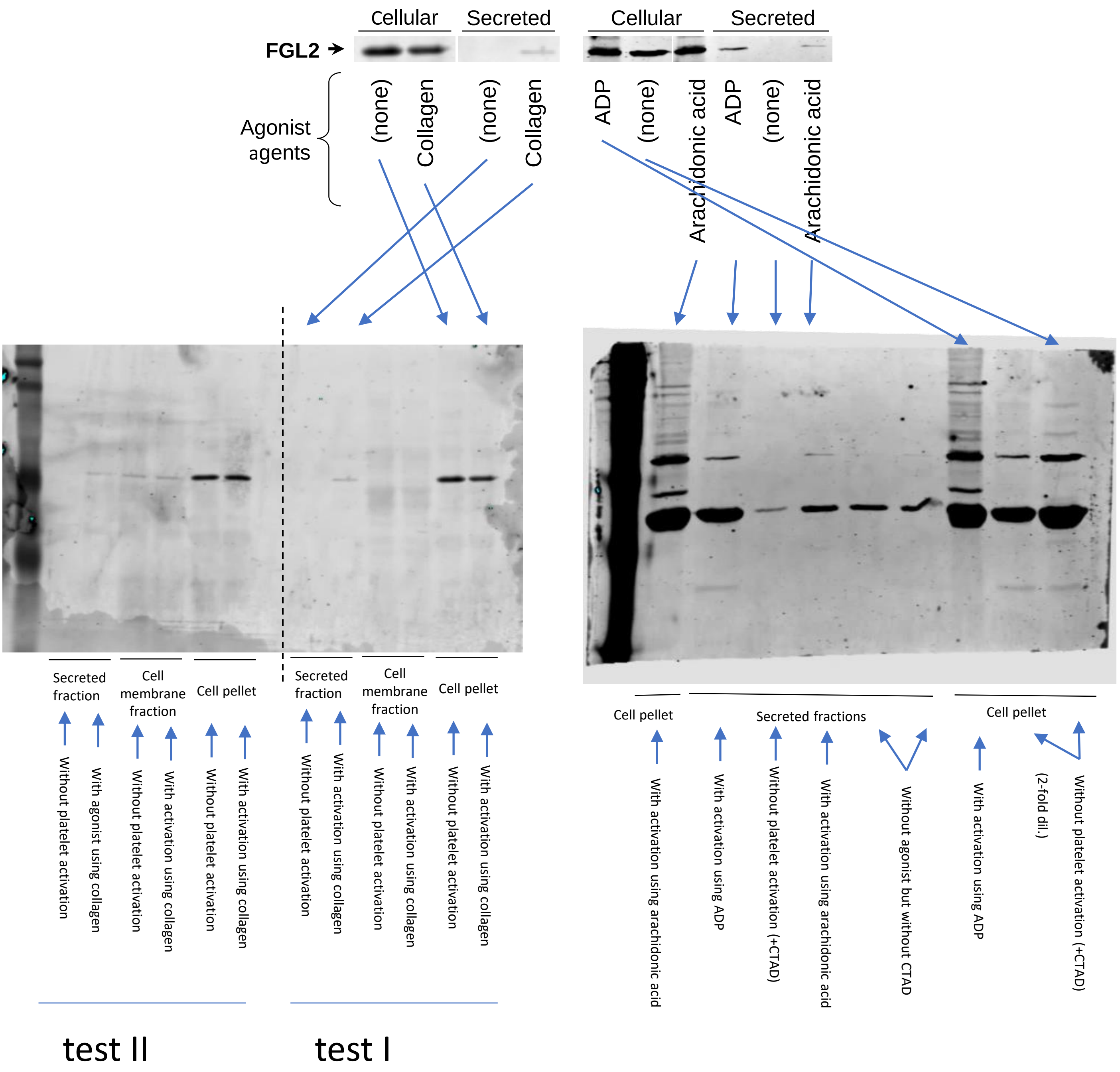
Cellular
Secreted
Cellular
Secreted
FGL2
Agonist agents
(none)
Collagen
(none)
Collagen
ADP
(none)
Arachidonic acid
ADP
(none)
Arachidonic acid
Secreted fraction
Cell membrane fraction
Cell pellet
Without platelet activation
With agonist using collagen
Without platelet activation
With activation using collagen
Without platelet activation
With activation using collagen
Secreted fraction
Cell membrane fraction
Cell pellet
Without platelet activation
With activation using collagen
Without platelet activation
With activation using collagen
Without platelet activation
With activation using collagen
Cell pellet
Secreted fractions
Cell pellet
With activation using archidonic acid
With activation using ADP
Without platelet activation (+CTAD)
With activation using arachidonic acid
Without agonist but without CTAD
With activation using ADP
(2-fold dil.)
Without platelet activation (+CTAD)
test II
test I
