# Supplementary material for: Presence and activity of Fibrinogen like protein 2 in platelets
Source: PLoS One. 2023 May 18;18(5):e0285735. doi: 10.1371/journal.pone.0285735 (PMC10194929; doi:10.1371/journal.pone.0285735)
Supplement: S1 File — (DOCX) [file pone.0285735.s005.docx]

**Supplementary Methods**

**FGL2 cloning and overexpression in PC3 cells**

The complete cDNA sequence of human Fgl2 (encoding for 439 aa protein) was inserted into a pCDNA3.1 vector (Invitrogen, USA) under a CMV promoter to provide constitutive overexpression. The construct was assembled using a restriction-free cloning technique. The Fgl2 fragment was amplified (using primers 5’-CTGTACGACGATGACGATAAGAAGCTGGCTAACTGGTACTGGC-3’ and 5’-CCACACTGGATCCTAGGTACTTATGGCTTAAAGTGCTTGGGTCTG-3’) and the vector fragment was amplified (using primers 5’-GTACCTAGGATCCAGTGTGG-3’ and 5’-CTTATCGTCATCGTCGTACAG-3’). Both reactions were treated with DpnI restriction enzyme (New England Biolabs, USA). The FGL2 and vector fragments were then allowed to anneal by slow temperature cool down. Annealed fragments were ligated using T4 ligase (New England Biolabs, USA) and amplified by PCR. All DNA amplification reactions were conducted using pfu Ultra II high fidelity DNA polymerase (Agilent Technologies, USA). Vectors carrying complete FGL2 were isolated and transfected into PC3 using Dharmafect-4 reagent (Dharmacon, USA) according to the manufacturer’s instructions. Stable clones harboring the FGL2 insert were obtained using neomycin 250 μg/ml G-418 (Promega, USA) as a selective marker. FGL2 overexpression was verified by real-time PCR and Western blotting at the transcription and translation levels, respectively.

**Western blotting**

Proteins were separated by SDS-PAGE (10% acrylamide gels) under reducing conditions, and blotted onto a nitrocellulose membrane. The human proteins FGL2 and factor-X were detected using the following primary antibodies: polyclonal rabbit anti-FGL2 IgG (11827-1-AP, Proteintech, USA); monoclonal mouse anti-FGL2 IgG_2a_ (M01, clone 6DT, Abnova, Taiwan; or sc-100276, Santa Cruz Biotechnology, USA); monoclonal mouse anti-factor-X IgG_1_ (sc-81739 Santa Cruz Biotechnology, USA). The following fluorophore-linked secondary antibodies were used: IRDye 680RD goat anti-mouse (Li-COR, USA) and IRDye 800CW goat anti-rabbit (LI-COR, USA). Immunoblots were imaged using an Odyssey imager and Image Studio Lite 5.0 software (LI-COR, USA).

**Enzyme-linked immunosorbent assay**

Plasma was obtained by centrifugation of peripheral blood samples at 4700 g, 25°C for 7 min. Platelet rich samples were obtained as described in the “platelets isolation” section above. Platelets samples included 1 × 10^8^ platelets. The levels of FGL2 or factor X were detected using enzyme linked immunosorbent assay (ELISA) kits (FGL2 kit - Biolegend, USA; Factor X kit - Assaypro, USA). All experiments were performed in duplicates according to the manufacturer's instructions. The proteins were quantified using a standard curve.
